# The Role of Computed Tomography and Real-Time Polymerase Chain Reaction in the Diagnosis and Prognostication of COVID-19

**DOI:** 10.7759/cureus.14424

**Published:** 2021-04-11

**Authors:** James T Roberts, Jaron M Hrushka, Samuel O Krider, Quan D Nguyen

**Affiliations:** 1 Diagnostic Radiology, University of Texas Medical Branch, Galveston, USA; 2 Neurological Surgery, University of Texas Medical Branch, Galveston, USA; 3 Radiology, University of Texas Medical Branch, Galveston, USA

**Keywords:** covid 19, chest x-ray (cx-ray), chest ct, screening, diagnosing, real-time pcr and nucleic acid sequencing, corona virus, imaging, imaging modalities

## Abstract

With the rapid spread of coronavirus disease 2019 (COVID-19) starting in early 2020, there has been much interest in the applicability of radiologic imaging in managing affected patients. From the initial screening to addressing the extent of pulmonary involvement, CT scans provide great value to hospitals overwhelmed by an influx of patients, including those with suspected COVID-19. Because CTs come at a high financial cost, lower cost real-time polymerase chain reaction (RT-PCR) COVID-19 tests are critical due to their ability to identify asymptomatic carriers and properly handle patients during the ongoing pandemic. However, unlike RT-PCR, CT scans can also provide insight into the progression of the virus. The signs of acute COVID-19 infection include unique patterns of ground-glass opacities (GGO) with vascular thickening, enabling radiologists to diagnose COVID-19 with a high specificity. Additionally, there may be a significant value in the use of CT scans in predicting the outcomes.

## Introduction

The rapid outbreak of the coronavirus disease 2019 (COVID-19) caused the World Health Organization to declare it a global pandemic in March 2020 [1]. Due to the emergence of acute respiratory distress syndrome (ARDS) within as few as six days from the onset of the initial symptoms of fever, dry cough, dyspnea, the early detection of COVID-19 is necessary for its proper management [2].

The value of real-time polymerase chain reaction (RT-PCR) tests for COVID-19 was initially controversial during the early spread because of its relatively low sensitivity, which was in the 60-70% range [3]. Although these RT-PCR tests gave high specificities, the low sensitivity would require multiple negative tests for disease exclusion. Moreover, during COVID-19's early spread in China, there was a shortage of RT-PCR kits, making low sensitivity tests impractical [3].

Chest CT scans for patients with suspected COVID-19 were initially thought to be highly sensitive but were considered unable to specify COVID-19 pneumonia [4]. Radiologists began to show that key characteristics such as peripheral distribution, ground-glass opacities (GGO), and vascular thickening lead to above 90% specificity in distinguishing COVID-19 pneumonia from other viral causes of pneumonia [4]. Radiologic characteristics in CT scans include the early presence of GGO frequently located in the inferior lobe or the right lung, followed by a progressive stage consisting of the spreading of GGO, and a peak stage where there is consolidation [5,6]. CT scans appear to be of great utility, especially in predicting the poor short-term outcomes of disease progression. Stratification of patients based on the risk of COVID-19 mortality can be possible in resource-limited hospitals so that more attention can be paid to patients with higher risks of mortality.

One early study stated that chest CT had a higher sensitivity of 97% than RT-PCR nasal swabs in detecting COVID-19 [5]. Furthermore, 60-93% of patients who had chest CT were labeled as positive and their symptoms were considered consistent with COVID-19 before positive RT-PCR readings [5]. One study also stated that chest CT had a greater sensitivity than RT-PCR and hence promoted the use of CT for diagnosing COVID-19 [7]. A more recent review article has argued that much of the previous literature that highlighted the efficacy of CT imaging for diagnosing COVID-19 was of low quality and required further analysis, stating that CT should be only used for detecting complications of COVID-19 pneumonia or for determining an alternative diagnosis instead of primarily diagnosing COVID-19 [8]. The following case demonstrates that CT scans in concordance with RT-PCR, clinical symptoms, and exposure history are associated with high utility in the screening and management of patients with possible COVID-19 infection, with CT scans being particularly valuable in providing insight into patient outcomes.

## Case presentation

A 61-year-old male with multiple comorbidities including diabetes, hypertension, and obstructive sleep apnea, which placed him in a “high risk” category for COVID-19, presented via telemedicine consultation with urgent care clinic with a fever that had started the previous day and cough. He reported experiencing a constant dry cough since undergoing intubation for hernia repair surgery last November. This first telemedicine visit led to a potential diagnosis of upper respiratory infection and he was determined to be low risk for COVID-19 with his current presentation of symptoms. The patient had traveled out of state to New Orleans two weeks prior to the presentation and was told to call urgent care if the fever recurred.

**Figure 1:**
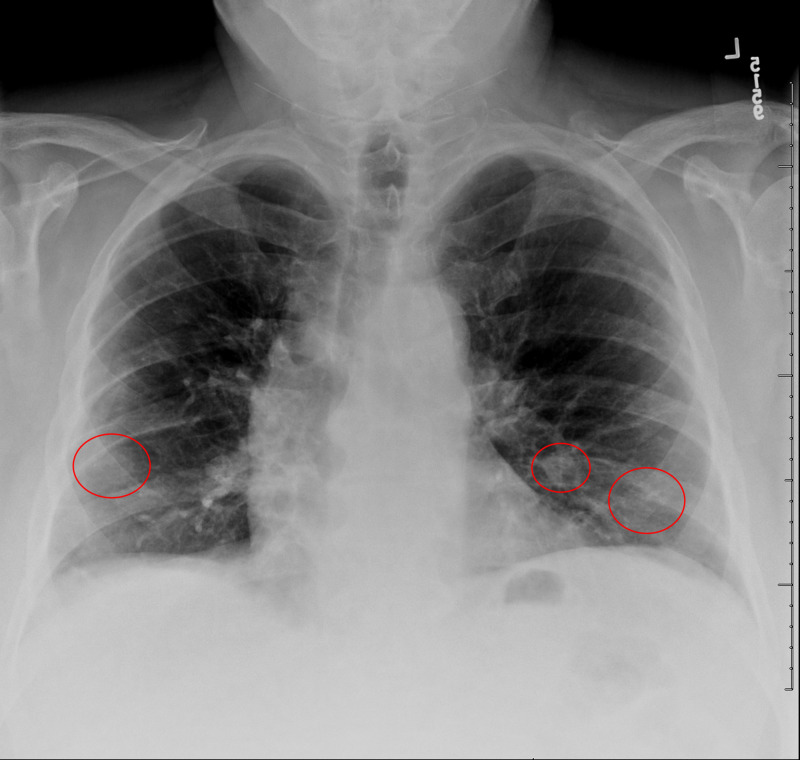
Chest X-ray of the patient Subtle hazy airspace opacities (red circles) in bilateral lower lungs suspicious for infections including COVID-19 pneumonia COVID-19: coronavirus disease 2019

**Figure 2:**
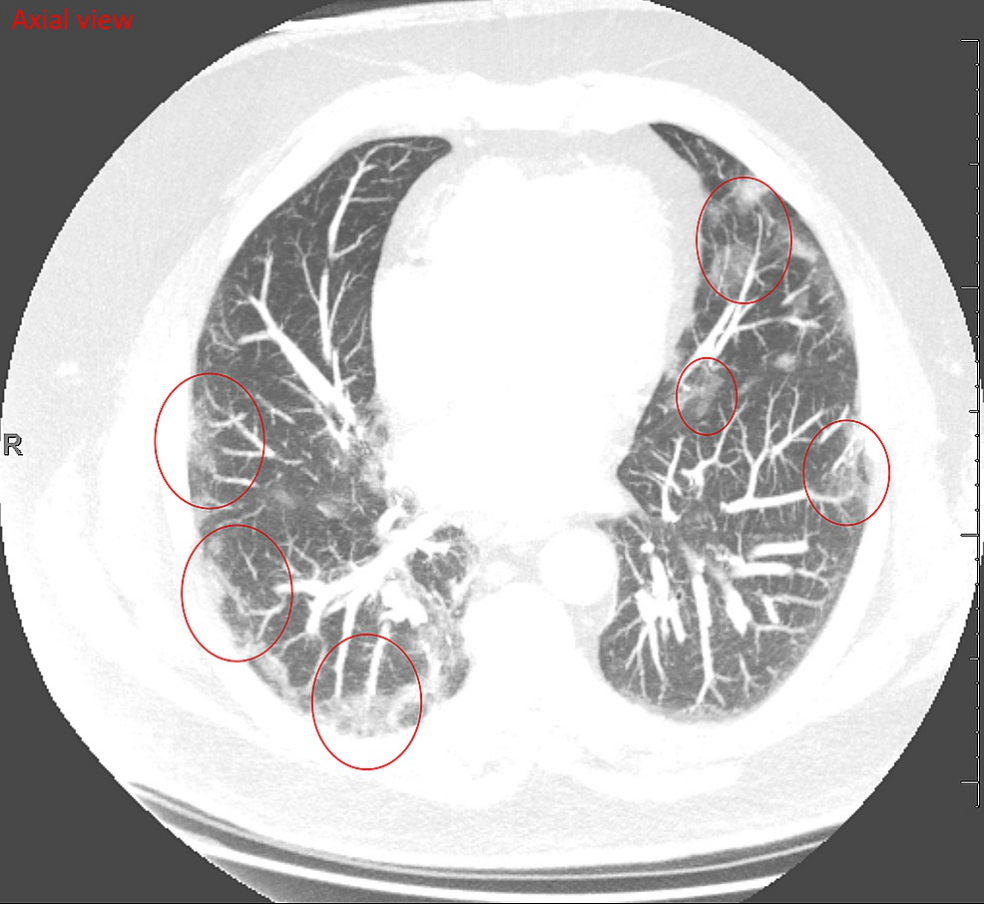
CT chest (axial view) Multilevel bilateral multifocal peripheral ground-glass opacities (red circles), suspicious for atypical pulmonary infection from bacterial and viral etiology, including COVID-19 CT: computed tomography; COVID-19: coronavirus disease 2019

**Figure 3:**
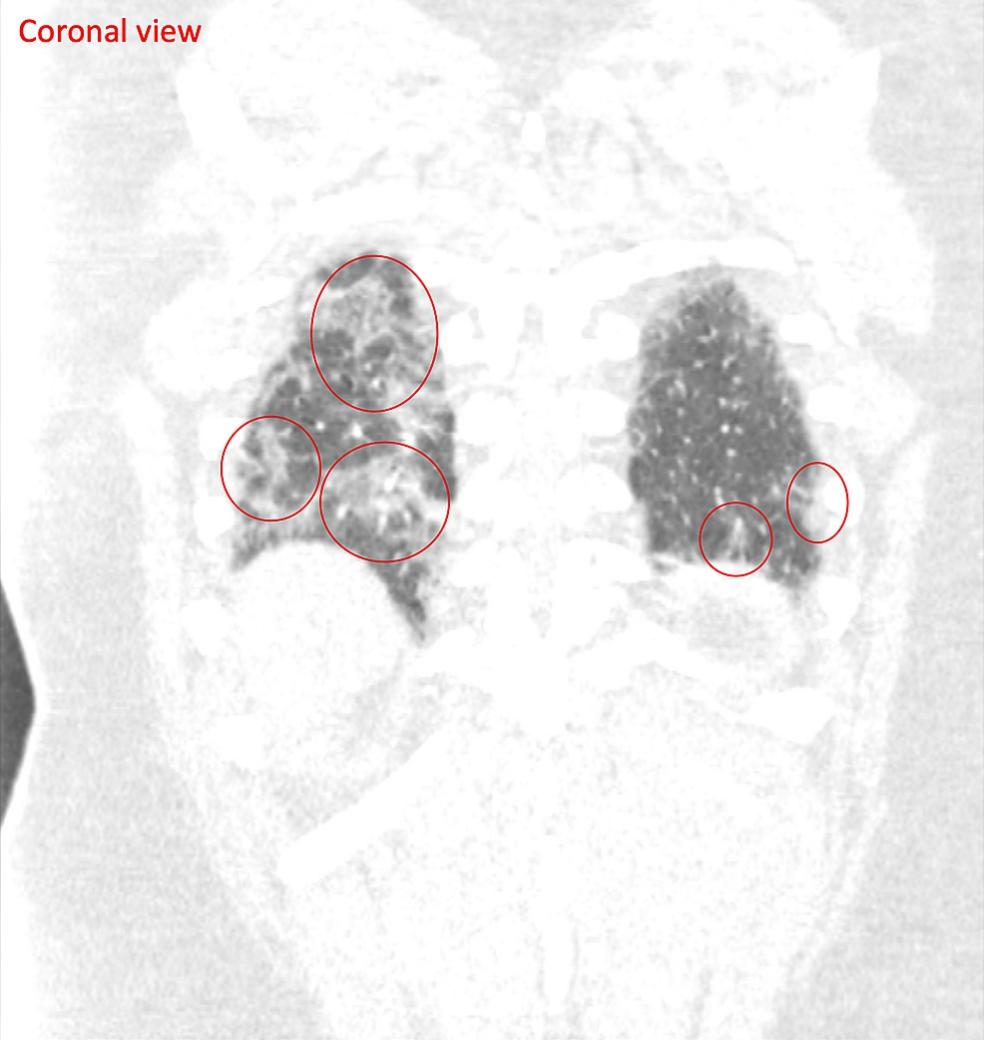
CT chest (coronal view) Multilevel bilateral multifocal peripheral ground-glass opacities (red circles), suspicious for atypical pulmonary infection from bacterial and viral etiology, including COVID-19 CT: computed tomography; COVID-19: coronavirus disease 2019

**Figure 4:**
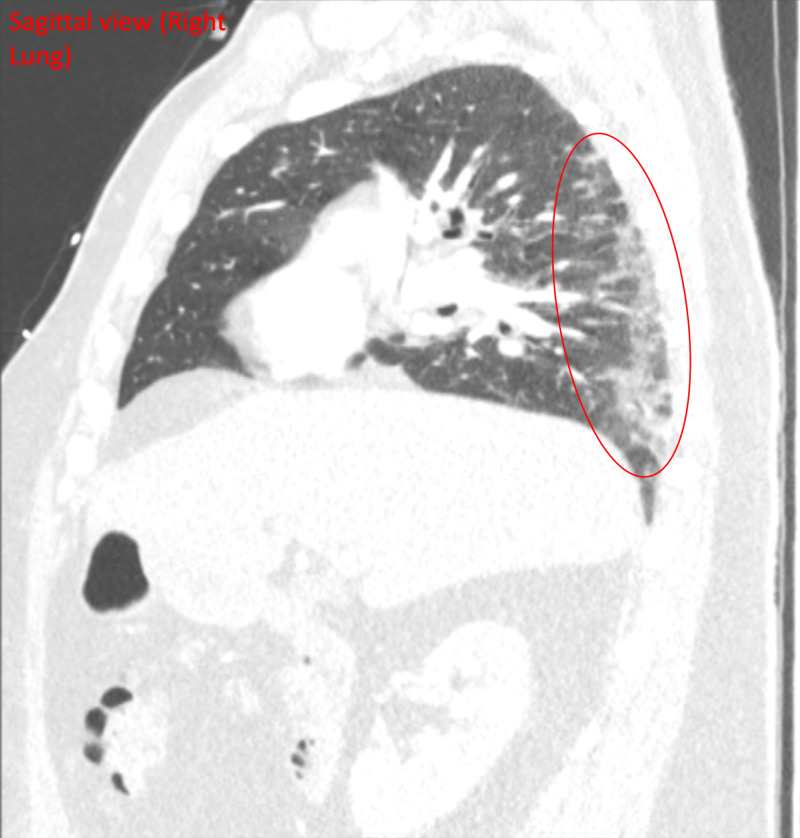
CT chest (sagittal view of the right lung) Multilevel bilateral multifocal peripheral ground-glass opacities (red circle), suspicious for atypical pulmonary infection from bacterial and viral etiology, including COVID-19 CT: computed tomography; COVID-19: coronavirus disease 2019

Two days after the initial telemedicine visit, the patient scheduled another telemedicine visit and reported a return of fever, productive cough, and shortness of breath. His fever had returned after his first telemedicine visit and had peaked at 101.5 °F, but he did not have a fever at the time of his second telemedicine visit. He also reported increasing shortness of breath and cough. He denied any sick contacts. The patient went to urgent care where he tested negative for flu. He was also swabbed for COVID-19 and given instructions to self-isolate while awaiting results. The patient also underwent a chest X-ray, which revealed subtle hazy opacities in bilateral lower lung zones, raising concerns related to infections including COVID-19 pneumonia (Figure 1); a chest CT demonstrated multilevel, peripheral GGO that raised concerns for an atypical lung infection from a virus or bacteria (Figures 2, 3, 4).

The patient was informed the day after the urgent care visit that he was COVID-19-positive and instructed to continue with self-isolation. He eventually had a full recovery.

## Discussion

A complete analysis of a patient with potential COVID-19 includes examining clinical symptoms, exposure history, testing, and if appropriate, imaging. The overall risk of a potential infection should be taken seriously; however, it may not be financially feasible for every patient who complains of a cough to receive a CT scan of the chest. The rapid test for COVID-19 has higher practical utility as it is faster and more cost-efficient than CT. The RT-PCR also has the advantage of detecting asymptomatic COVID-19 carriers and patients without respiratory findings on imaging.

There was much interest in the degree to which imaging can predict the COVID-19 progression as there are many self-reported anecdotal accounts of patients deteriorating in a short amount of time. Last May, researchers found that initial chest CT can predict poor short-term outcomes. Their study states that analyzing the extend of pulmonary abnormalities and finding consolidation with segmental vascular changes suggest poor short-term outcomes [9]. Thus, CT scans, particularly at the early stage of the pandemic when hospital beds and ventilators were in short supply, proved very valuable in identifying patients at risk of disease progression.

## Conclusions

RT-PCR in combination with examining clinical symptoms and exposure history appears to be the most common method of initial COVID-19 screening. Screening with CT scan is reserved for patients with acute respiratory distress or strong suspicion for COVID-19 infection. The CT scan has potential value in predicting the outcomes of the disease and could be used in medical settings with limited resources to identify patients who are more likely to deteriorate acutely. Currently, since more patients are potential carriers or have had COVID-19 in the past and may test positive on RT-PCR, chest CT could identify potential reinfection of COVID-19 if the immunity to the disease proves to wane over time.
